# Under-reporting of forensic findings: craniocervical emergency imaging in cases of survived hanging

**DOI:** 10.1007/s12024-023-00665-8

**Published:** 2023-06-20

**Authors:** J. Heimer, L. Arneberg, S. Blunier, J. Klukowska-Rötzler, A. G. Gonzenbach, A. Exadaktylos, T. Ruder, F. Wagner

**Affiliations:** 1 Department of Mathematics, Seminar for Statistics, ETH Zurich, Zurich, Switzerland; 2 Department of Emergency Medicine, Inselspital, Bern University Hospital and University of Bern, Bern, Switzerland; 3 Department of Medicine, Spital Emmental, Burgdorf, Switzerland; 4 Department of General Surgery, Spital Linth, Uznach, Switzerland; 5 Department of Diagnostic, Interventional and Pediatric Radiology, Inselspital, Bern University Hospital and University of Bern, Bern, Switzerland; 6 University Institute of Diagnostic and Interventional Neuroradiology, Inselspital, Bern University Hospital and University of Bern, Freiburgstrasse 10, 3010 Bern, Switzerland

**Keywords:** Nonfatal hanging, Underreported, Imaging findings, Clinical radiology, Forensic radiology

## Abstract

To determine the diagnostic bias between clinical and forensic radiology in cases of nonfatal hanging and determine and describe typical underreported imaging findings. In a retrospective, single-center study, all patients admitted for attempted suicide with near-hanging or fatal hanging between January 2008 and December 2020 who received CT or MRI of head and neck were reviewed and missed findings in the original report were documented. A binary regression with disagreement as dependent variable was fitted for the imaging modality, fatality, age, and sex. A total of 123 hanging incidents were retrospectively analyzed. The vast majority (*n* = 108; 87.8%) had attempted suicide with a nonfatal outcome. Fatal outcome occurred in 15 (12.0%). The extra- and intracranial injuries documented on CT and MRI scans were laryngeal (*n* = 8; 6.5%), soft tissue (*n* = 42; 34.1%), and vascular injuries (*n* = 1; 0.8%). Intracranial pathology was evident on 18 (14.6%) scans. Disagreement occurred in 36 (29.3%) cases and represented 52 (69.2%) of all cases with a radiological finding. Disagreement was strongly associated with fatality (OR: 2.7–44.9.4, *p* = 0.0012). In most cases, nonfatal hangings cause no or only minor injuries. Fatal cases are associated with a greater probability of missed minor imaging findings. This suggests that findings deemed clinically irrelevant are probably not reported in such severe emergency cases. This association indicates that minor abnormalities are underreported when major pathologies are evident on imaging in victims of strangulation.

## Introduction

“What distinguishes forensic pathologists from […] clinical radiologists is a focus on the end point of the forensic investigation,” wrote Stephen Cordner in the foreword to Brogdon’s *forensic radiology* [1]. Forensic and clinical radiology have fundamentally different goals: clinical radiology is a diagnostic tool that ensures the anatomical basis for therapy and the detection and staging of pathology; it is a forward-looking modality. Forensic radiology is motivated by causality. It looks exclusively into the past to determine the most likely cause of trauma. A potential dilemma arises from this fundamental difference: important forensic findings in clinical imaging may be overlooked due to being irrelevant for clinical radiology.

While (neuro-)radiologists read the images they obtain with a clinical motivation, forensic radiologists focus on findings important for medico-legal reconstructions and purposes, which are not necessarily important from a clinical point of view. Forensic radiologists are also able to explain certain phenomena visible on images due to their knowledge of thanatology.

Advanced forensic imaging is increasingly used in the courts and juries have responded positively to the presentation of forensic data. For these reasons, forensic imaging and image interpretation in the clinical context are becoming a regular part of forensic investigations. The increase in the use of forensic imaging presents a unique opportunity for (neuro-)radiologists to collaborate with forensic pathologists.

The existence of the diagnostic bias between clinical and forensic radiology is particularly interesting when studying the different forms of strangulation, which can be divided into hanging, ligature strangulation, and manual strangulation. In clinical emergencies, nonfatal strangulation (NFS) is an indication for contrast-enhanced craniocervical computed tomography (CT) to diagnose possible laryngeal injury and carotid dissection. Soft-tissue hematomas, which are clinically irrelevant, may also be detected during such an examination, but not reported. Such underreporting of forensic evidence represents a loss of information that could be relevant, especially for the forensic evaluation of NFS: in up to 50% of cases of NFS, patients show no external forensic findings [2]. Even fewer patients show injuries when only clinically relevant findings in alert patients are considered [3]. However, in the forensic assessment of NFS, additional radiologic findings can play a significant role [4–9].

A previous study [5] identified underreporting of forensic findings during clinical examinations in cases of manual strangulation. Clinical radiologists detected assault-related injuries in 21 of 114 cases (18.4%), whereas forensic radiologists detected such injuries in 49 (43.0%) of the cases. The present study aimed to further investigate this diagnostic bias in cases of strangulation, in this case hanging. It also sought to describe typical injuries observed in such cases to demonstrate the difficulty of their diagnosis in an emergency setting.

## Methodology

This study was a retrospective, longitudinal single-center study of all patients admitted for attempted suicide with near-hanging or fatal hanging to our Department of Emergency Medicine. It is a level 1 trauma center with 50,000 patients per year and a catchment area of 2 million inhabitants. Patients seen between January 2008 and December 2020 who received computed tomography (CT) or magnetic resonance imaging (MRI) of the head and neck were eligible.

In total, 123 patients, who had received an emergency craniocervical CT or MRI were selected from the medical report database of the hospital using the search terms: strangulation, nonfatal strangulation, hanging, CT, and MRI. The medical report of every hit in our computerized database (Ecare, Turnhout, Belgium) was then manually screened by one clinician to check whether the defined inclusion criterion of hanging plus an emergency CT or MRI scan was fulfilled. Patients who refused, or revoked general consent were excluded from the study.

Following the collection of eligible cases, one board-certified neuroradiologist (17 years expertise in radiology and 11 years expertise in neuroradiology; with European board head and neck certification) and one board-certified general radiologist (17 years of radiology experience with special focus on forensic radiology for 14 years) reviewed the images for: 1. signs of dissection of the cervical arteries, 2. injuries to the brain, 3. soft tissue, and 4. laryngeal cartilages. Both reviewers were blinded to the initial reporting performed during routine practice and had no knowledge of future reevaluation. The original and reevaluated reports were then compared, and any case of disagreement with regard to the four defined injury locations was documented.

The initial reports on the retrospectively analyzed patients were written by a resident in radiology training supervised by board-certified general radiologists or a resident in neuroradiology training supervised by board-certified neuroradiologists, depending on the body region examined. In the emergency setting the general radiologists are responsible for the neck and spine examinations whereas the neuroradiologists are in charge of the scans of the brain and extra- and intracranial vessels, independent of the modality (CT versus MRI).

## Ethical statement

This study was approved by the local ethics committee, followed the guidelines of the Declaration of Helsinki and ethical principles for conducting medical research with human subjects. No individual informed consent was obtained. All data were anonymized prior to analysis.

## Statistical analysis

To determine associations between disagreement and predictor variables, a generalized linear model with a logit link and a binomial likelihood (binary regression) with the occurrence of disagreement (binary) as the dependent, and age (continuous), sex (categorical), outcome (died or survived), and imaging modality (CT, MRI or both) was fitted. A significance level alpha of 0.05 was chosen in accordance with convention. Statistical analysis was performed using R statistical software (R Core Team) [10].

## Results

Table 1 shows the distribution of findings among the 123 patients who (temporarily) survived hanging, stratified by the presence of diagnostic disagreements (missed findings). The 123 near-hanging or fatal hanging incidents were analyzed. Most (*n* = 108; 87.8%) had attempted suicide, with nonfatal outcome. Fifteen cases (12.0%) were fatal. In 52 (42.3%) of cases, one or more radiological findings were present: 8 (6.5%) laryngeal injuries, 42 (34.1%) soft-tissue injuries, 18 (14.6%) intracranial injuries and one (0.8%) vascular injury.

**Table 1:** Summary of data stratified by the presence of radiological disagreements

**Level**		**No disagreement**	**Disagreement**
**Number (%)**		**87 (70.7)**	**36 (29.3)**
**Sex (%)**	Female	45 (51.7)	11 (30.6)
	Male	42 (48.3)	25 (69.4)
**Age in years (mean (SD))**		35.91 (15.43)	37.10 (14.18)
**Imaging (%)**	CT	65 (74.7)	30 (83.3)
	MRI	19 (21.8)	3 ( 8.3)
	CT and MRI	3 (3.4)	3 (8.3)
**Outcome (%)**	Survived	83 (95.4)	25 (69.4)
	Died	4 (4.6)	11 (30.6)
**Any (%)**	No injury	71 (81.6)	0 (0.0)
	Injury	16 (18.4)	36 (100.0)
**Larynx (%)**	No injury	80 (96.4)	30 (85.7)
	injury	3 (3.6)	5 (14.3)
**Superficial soft tissue (%)**	No injury	76 (89.4)	3 (8.3)
	Injury	9 (10.6)	33 (91.7)
**Vascular injury (%)**	No injury	81 (100)	35 (97.2)
	Injury	0 (0.0)	1 (2.8)
**Brain (%)**	No injury	60 (90.9)	18 (60.0)
	Injury	6 (9.1)	12 (40.0)

*CT* computed tomography, *MRI* magnetic resonance imaging

**Fig. 1:**
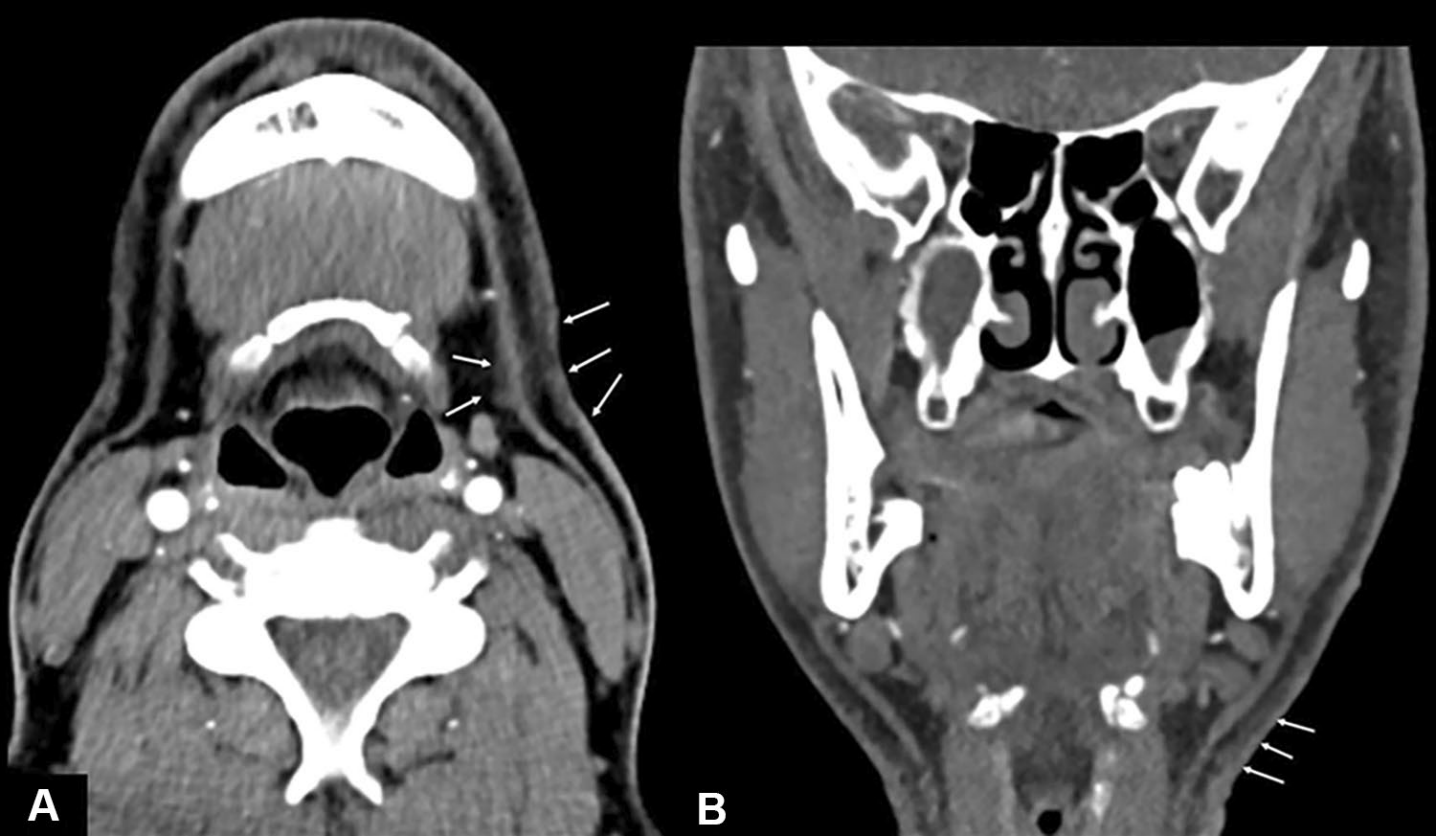
Axial and coronal non-enhanced CT (NECT); soft-tissue window with a slice thickness (ST) of 3 mm. The axial (**A**) and coronal (**B**) NECT images show a subtle stranding of the subcutaneous fat in the left submandibular region and a slight thickening of the cutis (arrows)

**Fig. 2:**
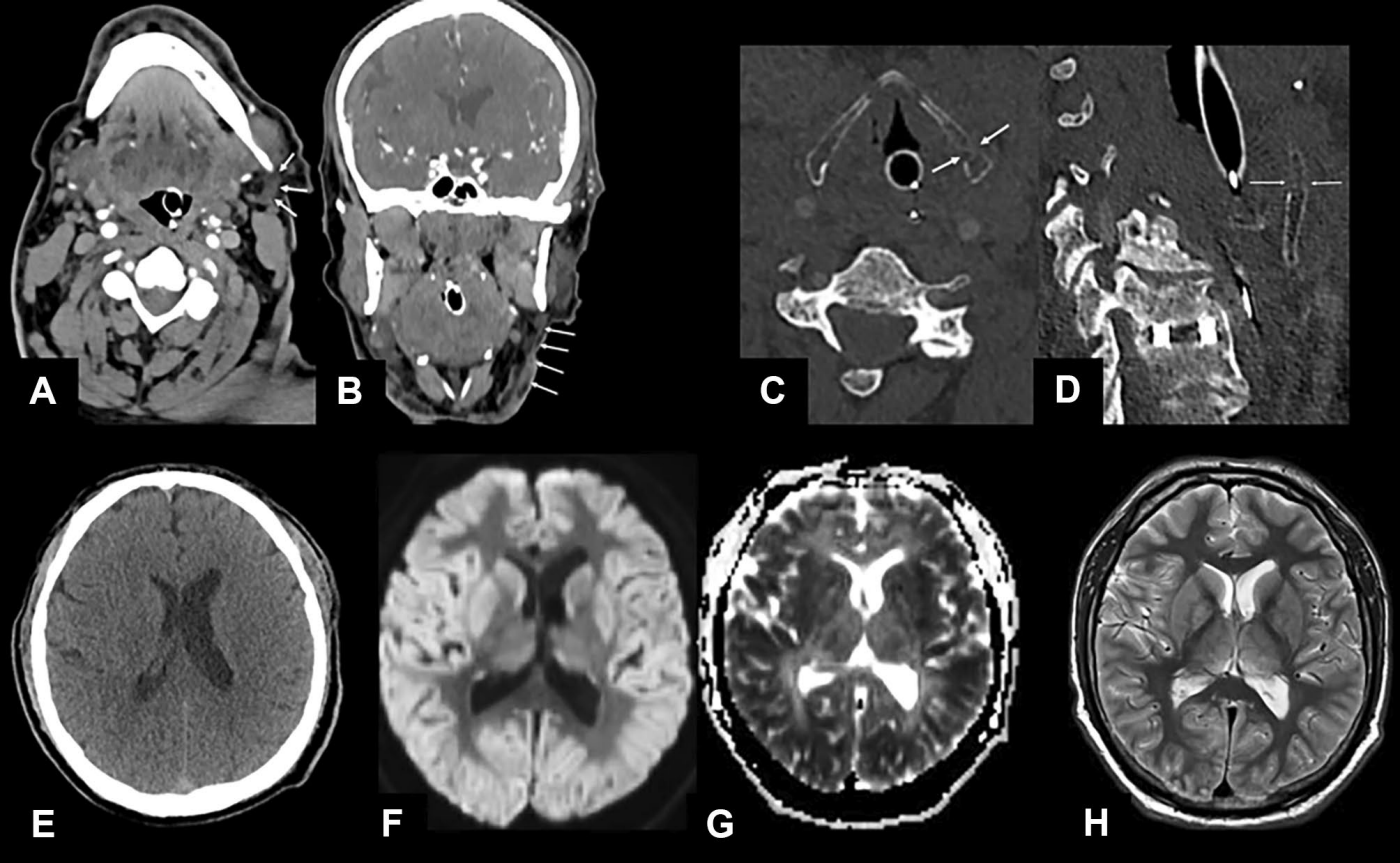
Axial and coronal NECT; soft tissue (3 mm ST) and bone window with a ST of 1 mm; axial and paracoronal slices. Non-enhanced CT and MRI of the brain with DWI (ST 3 mm) and ADC maps and a T2-weighted axial image (ST 3 mm). The axial (**A**) and coronal (**B**) NECT images reveal marked edema and stranding of the subcutaneous fat and cutis in the left submandibular region with edematous thickening of the cutis (arrows). The bone window of the larynx brought up a left-sided fracture of the thyroid cartilage with a clear kinking (arrows in **C** and **D**). On the non-enhanced emergency CT scan of the brain, diminished corticomedullary differentiation (**E**) is seen. The MRI scan of the brain (36 h after the initial event) confirmed the severe hypoxic-ischemic encephalopathy with diffusion restriction of the fronto-temporo-parieto-occipital cortex bi-hemispherically symmetric with involvement of the thalami and basal ganglia (**F** and **G**) with hyperintense demarcation on the T2-weighted image (**H**)

**Fig. 3:**
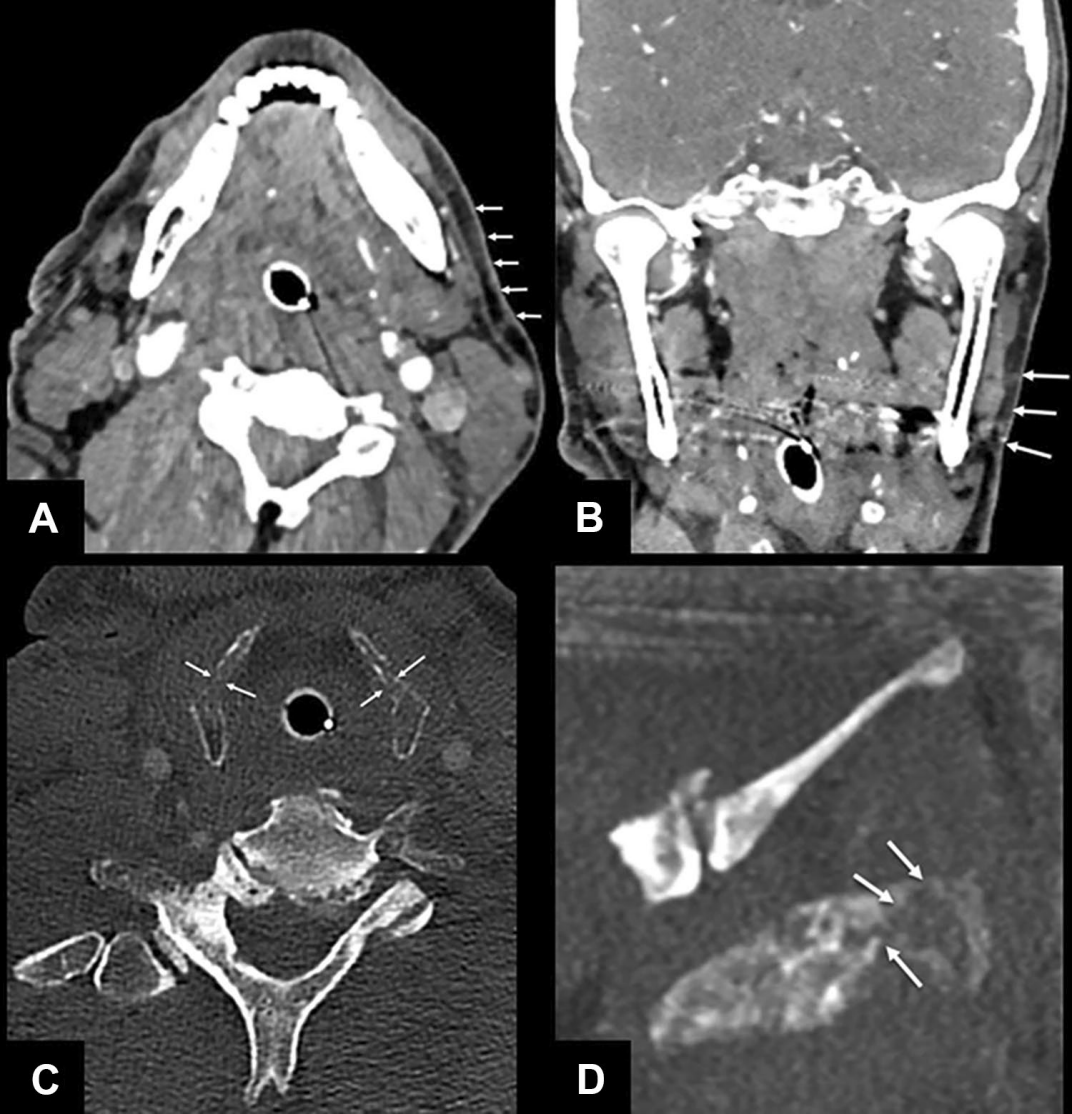
Axial and coronal NECT; soft tissue (3 mm ST) and bone window with a ST of 1 mm (axial and paracoronal image). The axial (**A**) and coronal (**B**) CT images demonstrate slight edema and stranding of the subcutaneous fat in the left perimandibular region with edematous thickening of the cutis (arrows). The bone window of the larynx brought up a bilateral fracture of the thyroid cartilage with buckling (arrows in **C** and **D**)

**Fig. 4:**
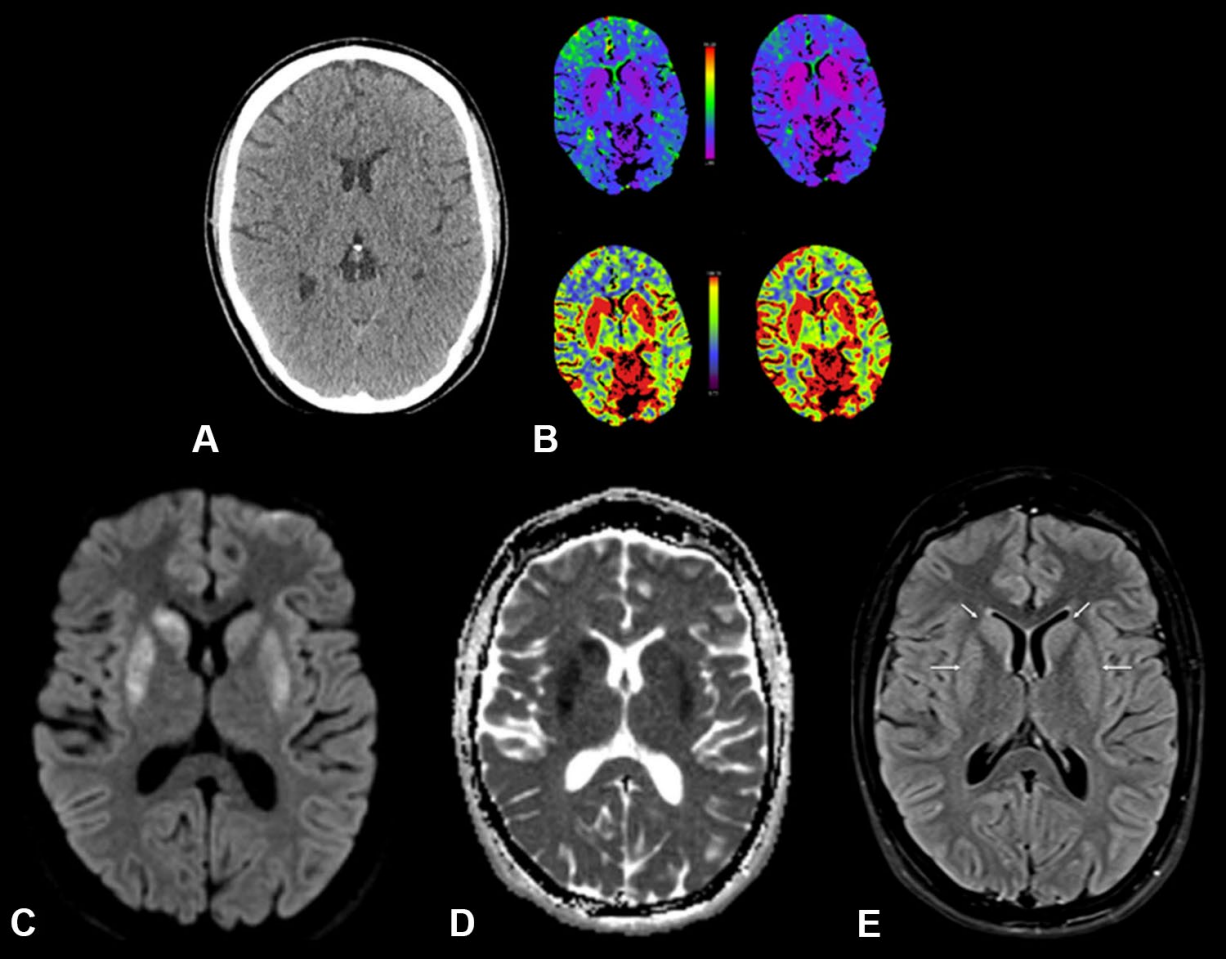
Axial NECT of the brain (ST 3 mm) with perfusion maps and MRI of the brain with DWI (ST 3 mm) and ADC maps and a FLAIR-weighted axial image (ST 4 mm). The axial native CT image of the brain shows diminished corticomedullary differentiation with involvement of the basal ganglia (**A**), confirmed in the CT perfusion maps (**B**). The MRI scan of the brain (28 h after the initial event) confirmed the severe hypoxic-ischemic encephalopathy with diffusion restriction of basal ganglia (arrows **C** and **D**) with demarcation on the FLAIR-weighted image (arrows in **E**)

**Fig. 5:**
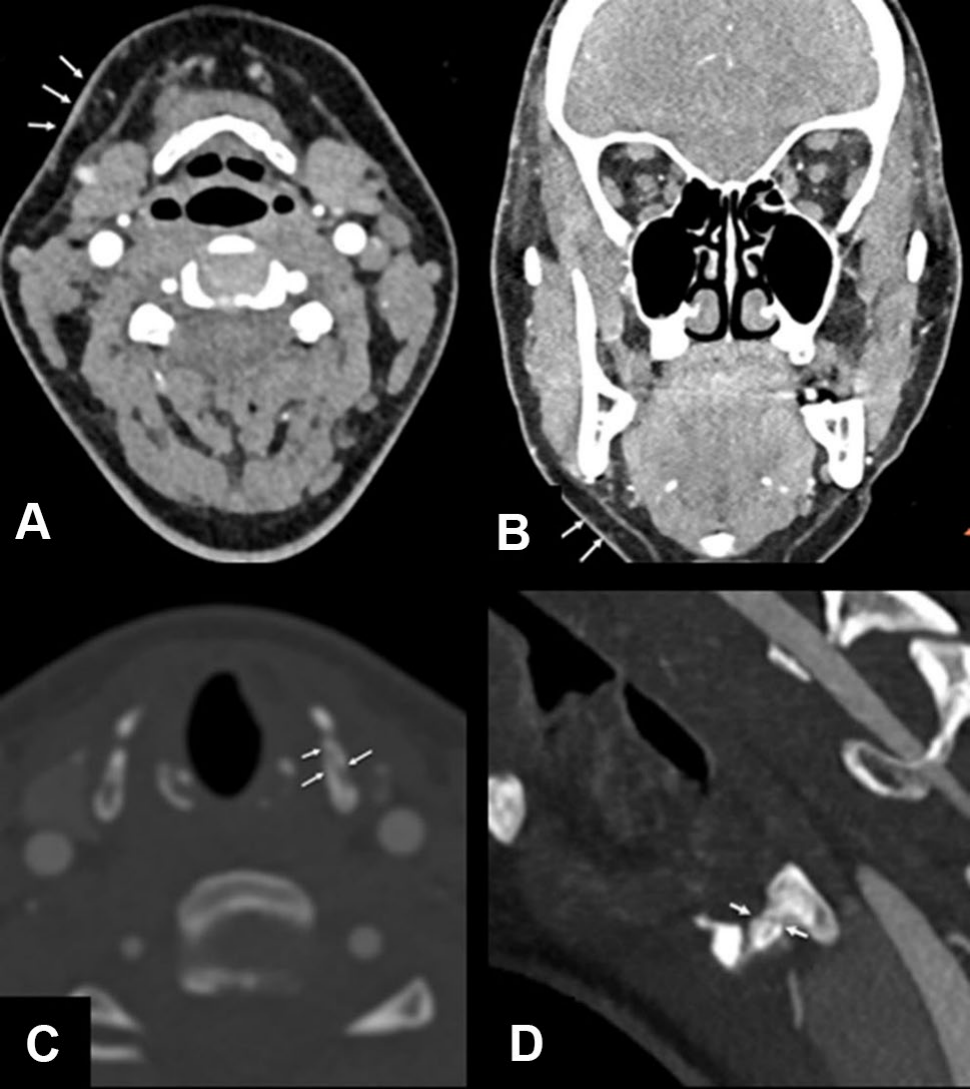
Axial and coronal NECT; soft tissue (3 mm ST) and bone window (axial and paracoronal) with a ST of 2 mm. The axial (**A**) and coronal (**B**) NECT images demonstrate slight edema and stranding of the subcutaneous fat in the right submental region with slight edematous thickening of the cutis (arrows). The bone window of the larynx brought up a left-sided undislocated fracture of the cricoid cartilage with buckling (arrows in **C** and **D**)

**Fig. 6:**
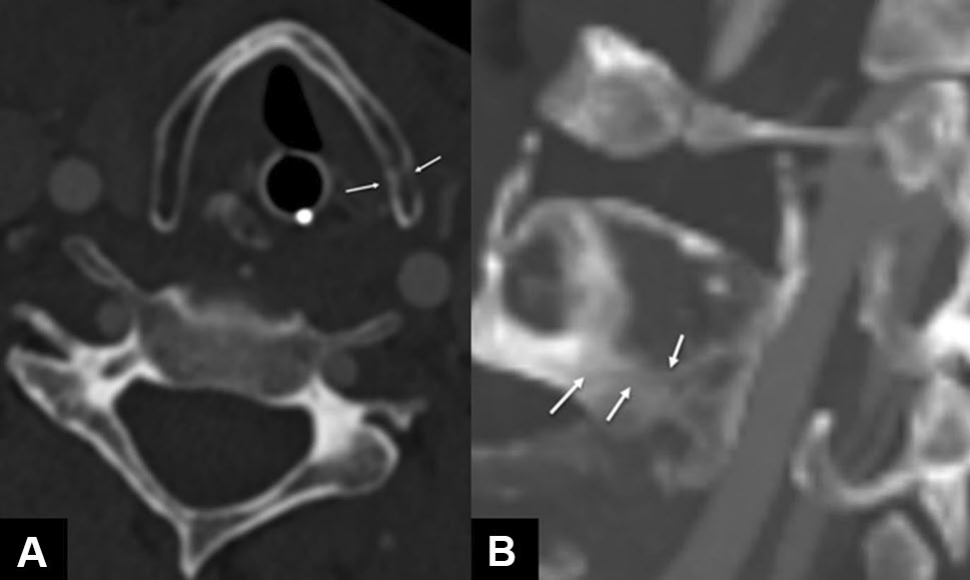
Axial and paracoronal NECT scan; bone window with a ST of 2 mm. The axial (**A**) and paracoronal (**B**) NECT images of the larynx reveal a left-sided subtle undislocated fracture line of the cricoid cartilage (arrows)

**Fig. 7:**
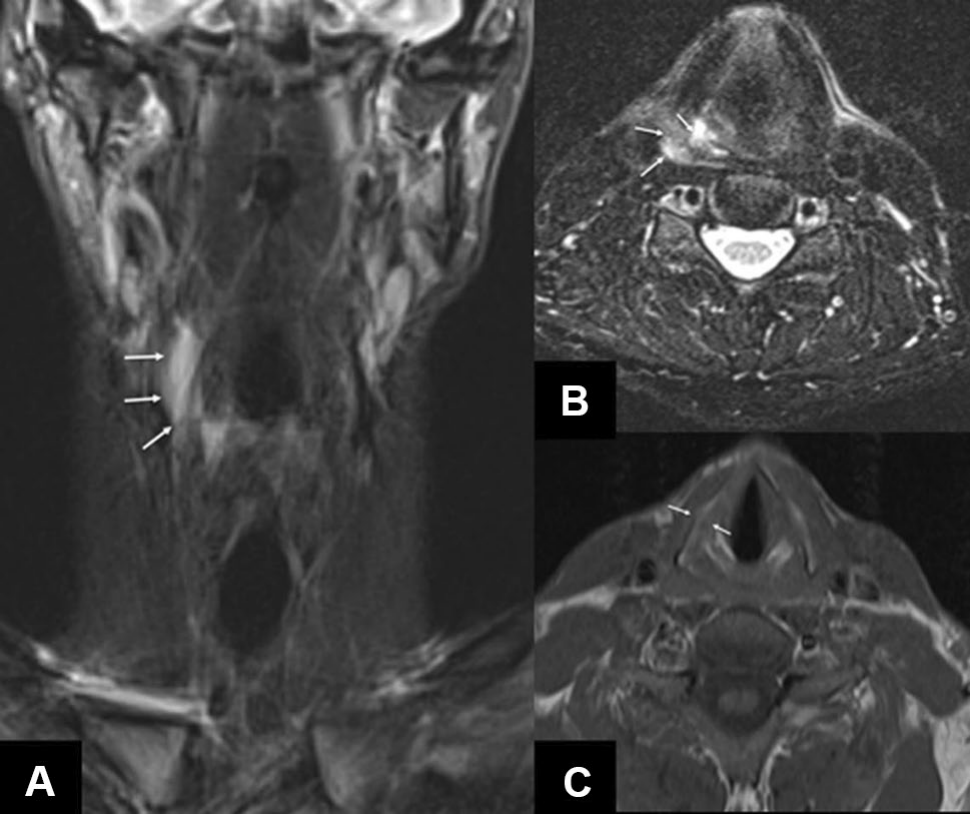
Non-enhanced MRI scan of the neck with a coronal STIR (ST 4 mm), an axial T2-weighted image with fat suppression (ST 3 mm) and a T1-weighted image (ST 3 mm). The MRI scan shows a marked hyperintense edema of the right-sided supraglottic larynx (arrows in **A** and **B**). The arrows in the T1-weighted image (**C**) point to the undislocated fracture of the left thyroid cartilage

**Fig. 8:**
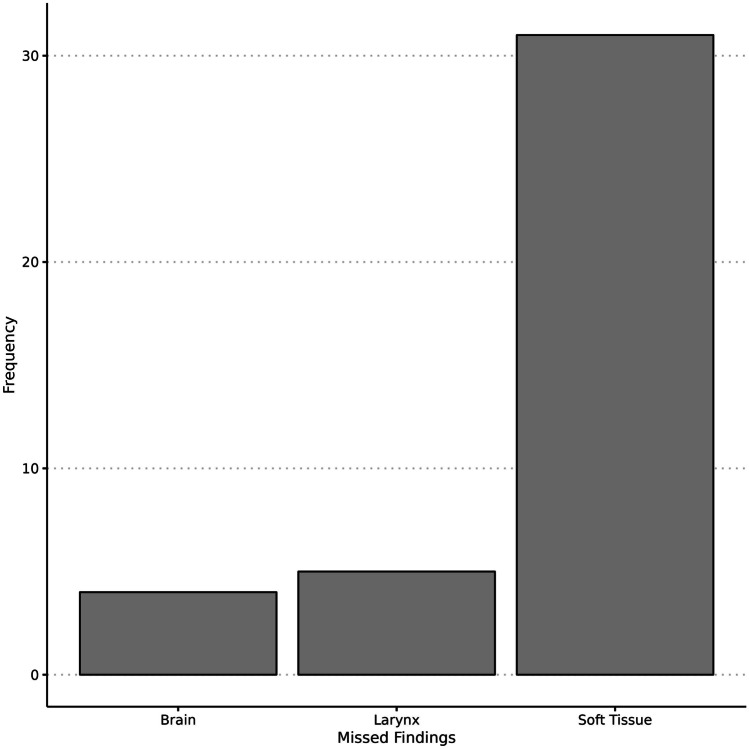
Bar plot of the distribution of disagreements by anatomical location of radiologic findings

Disagreement occurred in 36 (29.3%) cases, which represents 69.2% of all cases with a radiological finding (*n* = 52). Figures 1, 2, 3, 4, 5, 6, and 7 illustrate cases with missed findings and Fig. 8 shows the distribution of missed findings. The vast majority of missed disagreements were related to injuries to the soft tissue (31, 77.5%), followed by laryngeal injuries (5, 12.5%), and intracranial findings (4, 10.0%).

The odd ratios (OR) of the coefficients of predictor variables of the binary regression are shown in Table 2. The coefficients for sex, imaging modality, and age were not significantly associated with diagnostic disagreement. Disagreement was highly associated with later fatality from near-hanging (OR: 2.7–44.9.4, *p* = 0.0012).

**Table 2:** Overview of 95% confidence intervals of odds ratios of binary regression coefficients

			**2.50%**	**97.50%**
(Intercept)			0.1	1.42
Sex	**–**	**Male**	0.91	5.78
Outcome	**–**	**Fatal**	2.65	44.9
Age			0.95	1.01
Imaging	**–**	**MRI**	0.04	1.08
Imaging	**–**	**Both (CT and MRI)**	0.1	9.22

*CT* computed tomography, *MRI* magnetic resonance imaging

## Discussion

The purpose of this retrospective study was to report typical findings in cases of near-hanging and investigate possible underreporting of forensic findings in a cohort of survivors of attempted suicide by hanging.

Altogether, 123 hanging incidents were analyzed. The vast majority of patients had attempted suicide, with a nonfatal outcome. CT and MRI scans revealed the following extra- and intracranial injuries: laryngeal, soft-tissue, vascular, and intracranial pathologies. Disagreement occurred in 36 (29.3%) cases, and in 52 (69.2%) of all cases with a radiological finding. Disagreement was highly associated with fatality (*p* = 0.0012).

Our results imply that in most cases, nonfatal hangings will lead to no or only minor injuries, consistent with the literature. Relatively few cases of near-hanging show radiological findings [11]. This is similar to the findings in patients who survived manual strangulation, as summarized by Gascho et al. [4]. In cases of strangulation, the distribution of findings is highly dependent on the severity of the incident. Mild strangulation cases do not present with any findings, either externally or radiologically. In cases of fatality, the opposite is true, and all cases show external and radiological findings related to strangulation. Due to this duality, it is very difficult to define what constitutes life-threatening strangulation [12].

Conditional on the presence of a radiologic finding, in 69.2% of patients with radiological findings, forensic findings were underreported. Several reasons why these findings were underreported are possible.

Disagreement was strongly associated with fatality. Likely fatal outcome is predicted by the clinical radiologist as, for example, severe brain edema on the images. It is not surprising that a clinical radiologist would not specifically look for mild injury to the soft tissue when confronted with a very likely fatal outcome for a patient, without being directly prompted to do so.

Similarly, this imaging is performed in an emergency setting. In this setting, the central need is to rapidly exclude the possibility of (treatable) imminent death—i.e., an upper airway obstruction due to laryngeal or tracheal compression, or the presence of a carotid artery dissection. This entails a focus on important findings, while minuscule trauma not connected to the emergency diagnosis is not reported. Within the categories of bias in radiology coined by Busby et al. [13], this underreporting would come under “satisfaction of search,” as the important, clinical questions have been answered.

Thirdly, underreporting is common in settings in which the theoretical probability of a radiological finding, such as reported for cases of near hangings, is not very high [11]. This means, that a radiologist already has the hypothesis that there will be no important findings, an example of “confirmations bias” [13].

The very high proportion of underreporting in cases of near-hanging indicates that a forensic assessment of clinical radiologic imaging should always be followed by a second forensic radiological review to serve as evidence in forensic pathology. The results further indicate that forensic and clinical radiologists have fundamentally different perceptions of the same imaging material, which leads to differences between the forensic and clinical radiological reports.

At present, there is no authorized path to becoming a board-certified forensic radiologist in Europe. The International Society of Forensic Radiology and Imaging unites the interests of this growing subdiscipline (https://www.isfri.org/). Currently, a few universities offer continuing education programs in forensic radiology, such as the virtopsy course and the Certificate of Advanced Studies in Forensic Imaging and Virtopsy at the Forensic Institute at the University of Zurich, Switzerland. Other examples are the MSc program “Post-Mortem Radiology for Natural and Forensic Death Investigation” at the University of Leicester (UK) and the “Post Mortem CT Interpretation Short Course” at Monash University in Melbourne, Australia. The European Society of Radiology offers a 90-min online forensic radiology course: Radiology of the Afterlife (https://connect.myesr.org/course/adult-postmortem-imaging/). We believe that especially radiologists and neuroradiologists in emergency services should be better trained in forensic radiology in the future. Forensic radiology should be an integral part of the general radiology curriculum.

This retrospective study has some limitations. First, the overall number of patients studied was relatively small, limiting statistical analysis. Second, we are aware that the interpretation of a craniocervical CT or MRI scans in an emergency setting depends on the experience of the reader. Both study raters have long-standing experience in emergency radiology and might be more aware of subtle imaging findings. Finally, the retrospective nature of this study and the fact that images were acquired in clinical emergency settings over a period of 12 years, during which protocols and scanners were upgraded, means that the data consist of CT scans and MRI sequences obtained using varying scanning parameters and protocols.

## Conclusion

Similar to manual strangulation, a large proportion of cases of nonfatal hanging do not present with any radiologic findings. If findings are present, they are mostly soft-tissue hematomas and laryngeal fractures. This study revealed statistically significant underreporting of forensic findings during emergency craniocervical CT and/or MRI in victims of strangulation. This underreporting is associated with fatality of the attempted hanging, which indicates that less severe injuries are likely not considered important or clinically relevant in the emergency setting.

The combination of scarce findings in all modalities of NFS and increasing evidence for clinical underreporting of forensic findings indicates a need for a forensic review of radiologic images, particularly if they are to serve as evidence in forensic pathology.

## Key points

1. Most cases of nonfatal hanging cause no or only minor injuries.
2. Missed imaging findings are strongly associated with a bad outcome prognosis, indicating that these missed imaging findings are clinically irrelevant in the emergency setting.
3. The scarce findings in cases of nonfatal hanging, combined with increasing evidence for clinical underreporting of forensic findings, suggests a need for a forensic review of radiologic images in nonfatal hanging.

## Data Availability

Data are available upon reasonable request.

## Abbreviations

NFS: Nonfatal strangulation
CT: Computed tomography
NECT: Non-enhanced computed tomography
MRI: Magnetic resonance imaging
ST: Slice thickness
OR: Odds ratio

## References

[1] Michael J. Thali MDV, B. G. Brogdon. Brogdon’s forensic radiology second ed. Boca Raton: CRC Press; 2010.

[2] Strack GB, McClane GE, Hawley D. A review of 300 attempted strangulation cases. Part I: criminal legal issues. J Emerg Med. 2001;21(3):303–9. 10.1016/s0736-4679(01)00399-7. 10.1016/s0736-4679(01)00399-7 11604294

[3] Matusz EC, Schaffer JT, Bachmeier BA, Kirschner JM, Musey PI Jr, Roumpf SK, et al. Evaluation of nonfatal strangulation in alert adults. Ann Emerg Med. 2020;75(3):329–38. 10.1016/j.annemergmed.2019.07.018. 31591013 10.1016/j.annemergmed.2019.07.018

[4] Gascho D, Heimer J, Tappero C, Schaerli S. Relevant findings on postmortem CT and postmortem MRI in hanging, ligature strangulation and manual strangulation and their additional value compared to autopsy - a systematic review. Forensic Sci Med Pathol. 2019;15(1):84–92. 10.1007/s12024-018-0070-z. 30627977 10.1007/s12024-018-0070-z

[5] Heimer J, Tappero C, Gascho D, Flach P, Ruder TD, Thali MJ, et al. Value of 3T craniocervical magnetic resonance imaging following nonfatal strangulation. Eur Radiol. 2019;29(7):3458–66. 10.1007/s00330-019-06033-x. 30796576 10.1007/s00330-019-06033-x

[6] Yen K, Thali MJ, Aghayev E, Jackowski C, Schweitzer W, Boesch C, et al. Strangulation signs: initial correlation of MRI, MSCT, and forensic neck findings. J Magn Reson Imaging. 2005;22(4):501–10. 10.1002/jmri.20396. 16142698 10.1002/jmri.20396

[7] Yen K, Vock P, Christe A, Scheurer E, Plattner T, Schön C, et al. Clinical forensic radiology in strangulation victims: forensic expertise based on magnetic resonance imaging (MRI) findings. Int J Legal Med. 2007;121(2):115–23. 10.1007/s00414-006-0121-y. 17206435 10.1007/s00414-006-0121-y

[8] Bruguier C, Genet P, Zerlauth JB, Dédouit F, Grimm J, Meuli R, et al. Neck-MRI experience for investigation of survived strangulation victims. Forensic Sci Res. 2020;5(2):113–8. 10.1080/20961790.2019.1592314. 32939427 10.1080/20961790.2019.1592314 PMC7476612

[9] Christe A, Thoeny H, Ross S, Spendlove D, Tshering D, Bolliger S, et al. Life-threatening versus non-life-threatening manual strangulation: are there appropriate criteria for MR imaging of the neck? Eur Radiol. 2009;19(8):1882–9. 10.1007/s00330-009-1353-2. 19283386 10.1007/s00330-009-1353-2

[10] R Core Team. R: A language and environment for statistical computing. R Foundation for Statistical Computing, Vienna, Austria. 2019. https://www.R-project.org/.

[11] Ribaute C, Darcourt J, Patsoura S, Ferrier M, Meluchova Z, Gramada R, et al. Should CT angiography of the supra-aortic arteries be performed systematically following attempted suicide by hanging? J Neuroradiol. 2021;48(4):271–6. 10.1016/j.neurad.2019.04.001. 31034897 10.1016/j.neurad.2019.04.001

[12] Plattner T, Bolliger S, Zollinger U. Forensic assessment of survived strangulation. Forensic Sci Int. 2005;153(2–3):202–7. 10.1016/j.forsciint.2004.09.106. 16139111 10.1016/j.forsciint.2004.09.106

[13] Busby LP, Courtier JL, Glastonbury CM. Bias in radiology: the how and why of misses and misinterpretations. Radiographics. 2018;38(1):236–47. 10.1148/rg.2018170107. 29194009 10.1148/rg.2018170107 PMC5790309

